# Both flagella and F4 fimbriae from F4ac^+^ enterotoxigenic *Escherichia coli* contribute to attachment to IPEC-J2 cells in vitro

**DOI:** 10.1186/1297-9716-44-30

**Published:** 2013-05-13

**Authors:** Mingxu Zhou, Qiangde Duan, Xiaofang Zhu, Zhiyan Guo, Yinchau Li, Philip R Hardwidge, Guoqiang Zhu

**Affiliations:** 1 College of Veterinary Medicine, Yangzhou University, Yangzhou, 225009, China; 2 Agriculture college, Weinan Vocational and Technical College, Weinan, 714000, China; 3 Department of Dermatology of Clinical Medical School, Yangzhou University, Yangzhou, 225009, China; 4 College of Veterinary Medicine, Kansas State University, Manhattan, KS, 66506, USA

## Abstract

The role of flagella in the pathogenesis of F4ac^+^ Enterotoxigenic *Escherichia coli* (ETEC) mediated neonatal and post-weaning diarrhea (PWD) is not currently understood. We targeted the reference C83902 ETEC strain (O8:H19:F4ac^+^ LT^+^ STa^+^ STb^+^), to construct isogenic mutants in the *fliC* (encoding the major flagellin protein), *motA* (encoding the flagella motor), and *faeG* (encoding the major subunit of F4 fimbriae) genes. Both the Δ*fliC* and Δ*faeG* mutants had a reduced ability to adhere to porcine intestinal epithelial IPEC-J2 cells. F4 fimbriae expression was significantly down-regulated after deleting *fliC*, which revealed that co-regulation exists between flagella and F4 fimbriae. However, there was no difference in adhesion between the Δ*motA* mutant and its parent strain. These data demonstrate that both flagella and F4 fimbriae are required for efficient F4ac^+^ ETEC adhesion in vitro.

## Introduction

ETEC is a major cause of diarrhea in neonatal and young pigs, causing significant economic losses, treatment costs, and reduced production efficiency. The key virulence factors of ETEC-mediated diarrhea include (i) adhesins, which mediate bacterial attachment to host enterocytes and initiate *E. coli* colonization and (ii) enterotoxins, which disrupt fluid homeostasis in the host small intestine and cause fluid hyper-secretion. ETEC strains expressing F4 (K88) fimbriae, heat-labile enterotoxin (LT), and heat-stable enterotoxin (ST) are highly prevalent. Previous cell culture studies showed that exclusion of F4^+^ ETEC from attachment to epithelial cells requires repression of both the adhesin and LT [1].

Flagella have been generally regarded as a virulence factor, mainly because of their locomotive properties. As more information has been gathered, it is now known that this organelle participates in many additional processes including adhesion, biofilm formation, virulence factor secretion, and the modulation of the immune system of eukaryotic cells [2-6]. Our recent work demonstrated that flagella in F18ab^+^ Shiga-toxin producing *E. coli* (STEC) act as virulence factors that contribute to adhering to and invading IPEC-J2 cells, forming biofilms, and stimulating IL-8 production from Caco-2 cells in vitro [7].

The purpose of this study was to investigate whether flagella in F4^+^ ETEC have similar functions. We deleted the *fliC* gene (encoding the major flagellin protein), the *motA* gene (encoding the flagella motor), and the *faeG* gene (encoding the major subunit of F4 fimbriae) in the wild-type strain C83902 (O8:H19:F4ac^+^ LT^+^ STa^+^ STb^+^). Using the undifferentiated piglet jejunum intestinal epithelial cell line IPEC-J2 as an in vitro cell model, we demonstrate that the flagellum of F4ac^+^ ETEC is an important virulence factor, acting in concert with F4 fimbriae, in adhering to IPEC-J2 cells in vitro.

## Materials and methods

### Bacterial strains and culture conditions

*E. coli* C83902 (O8:F4ac^+^, LT^+^, STa^+^, STb^+^) parent strain and the isogenic mutants C83902 Δ*fliC*, C83902 Δ*motA*, C83902 Δ*faeG*, and double mutants C83902 Δ*fliC* Δ*faeG* were grown in LB broth or on LB agar plates at 37°C, in the presence of ampicillin (100 μg/mL) or chloramphenicol (34 μg/mL) where appropriate.

### Cell line culture conditions

Porcine neonatal jejunal epithelial cell line IPEC-J2 cells [8] were cultured in antibiotic free F12- RPMI1640 (1:1) mixed media (Gibco, NY, USA), supplemented with 10% new-born calf serum (NCS) (Gibco, NY, USA). The cells were maintained in 75 mL flasks (Corning, NY, USA) at 37°C in a humidified incubator in an atmosphere of 5% CO_2_.

### Construction of the isogenic mutants for C83902 *E. coli* and the complemented strain Δ*fliC/*p*fliC*

The isogenic C83902 mutants were generated using the λ-Red recombinase as described previously [9]. Using *fliC* as an example, primers homologous to sequences within the 5′ and 3′ regions of *fliC* were designed to replace the *fliC* gene with sequences carrying the chloramphenicol resistance-encoding gene cassette derived from the template plasmid pKD3 by PCR amplification (Δ*fliC*-Cm-F, Δ*fliC*-Cm-R) (Table 1). Gel-purified PCR products were electroporated into C83902 containing plasmid pKD46. LB plates containing both chloramphenicol and ampicillin were used to select the primary recombinants. The Flp recombinase-expressing vector pCP20 was used to excise the chloramphenicol cassette. The final mutant of *fliC* deletion in the C83902 strain was confirmed by both PCR screening with *fliC*-specific primers (*fliC*-F, *fliC*-R) (Table 1) and by DNA sequencing. The Δ*motA*, Δ*faeG* and the Δ*fliC* Δ*faeG* double mutant were constructed in a similar way. Full-length *fliC* gene was amplified by PCR from C83902 genomic DNA to generate a complementation strain. PCR primers are listed in Table 1. The expected 1.8 kbp length of the *fliC* gene were inserted into the pBR322 vector, and confirmed by combined DNA sequencing and restriction enzyme digestion. The recombinant pBR322 plasmid was then introduced into the C83902 Δ*fliC* strain.

**Table 1:** Primers used in this study

**Primer**	**Sequences (5′-3′)**
Δ*fliC*-Cm-F	TATCGAGCGTCTGTCTTCTGGCTTGCGTATTAACAGCGCGAAGGATGACGTGTGTAGGCTGGAGCTGCTTCG
Δ*fliC*-Cm-R	ACGGGACTGCGCTTCRGACAGGTTGGTAGTGGTGTTGTTCAGGTTGGTCATATGAATATCCTCCTTAG
Δ*faeG*-Cm-F	ATTTCAATGGTTCGGTCGATATCGGTGGTAGTATCACTGCAGATGATTATTGTGTAGGCTGGAGCTGCTTCG
Δ*faeG*-Cm-R	AGTTACAGCCTGATTAAAAGTTGCCTCAATAGTCTGACCGTTTGCAATCATATGAATATCCTCCTTAG
Δ*motA*-Cm-F	TCGGTACAGTTTTCGGCGGTTATTTGATGACCGGTGGAAGCCTTGGAGCATGTGTAGGCTGGAGCTGCTTCG
Δ*motA*-Cm-R	CGACGGACGTTCGCTGGAATAGAGCGTTTTGCGACCAAACTCAACGGCCATATGAATATCCTCCTTAG
*fliC*-F	ATGGCACAAGTCATTAATACCAACA
*fliC*-R	TTAACCCTGCAGCAGAGACAGA
*faeG*-F	ATGAAAAAGACTCTGATTGCACTG
*faeG*-R	TTAGTAATAAGTAATTGCTACGTTC
*motA*-F	GTGCTTATCTTATTAGGTTACCTGGTTGT
*motA*-R	TCATGCTTCCTCGGTTGTCGT
pBR-*fliC*-F	GCTCTAGAATGGCACAAGTCATTAATACCAACAG
pBR-*fliC*-R	CAGCGTCGACTTAACCCTGCAGCAGAGACAGAAC
*gapA*-RT-F	CGTTAAAGGCGCTAACTTCG
*gapA*-RT-R	ACGGTGGTCATCAGACCTTC
*faeG*-RT-F	ACTCAGAAAACCTGATGGTGAAACT
*faeG*-RT-R	CCCCACCTCTCCCTAACACA
*fliC*-RT-F	TCGACAAATTCCGCTCCTC
*fliC*-RT-R	GGTTGGTGGTGGTGTTGTTC

### Transmission electron microscopy

Flagella morphology of the C83902 strain and isogenic mutants were examined using transmission electron microscopy (TEM). After being negatively stained with 1% phosphotungstic acid (pH 7.4) for 2 min on carbon–formvar copper grids, samples were examined using a Philips CM-100 transmission electron microscope at 60 kV.

### Motility assays

Motility assays were performed as described previously [10]. Briefly, 50 μL of an overnight culture was re-inoculated into 5 mL of sterile LB broth and incubated at 37°C with aeration (180 rpm) until the value of approximately 1.0 at OD_600_ was achieved to indicate a logarithmic phase. One microliter of each bacteria culture was dropped onto semisolid agar plates (Tryptone 1%, NaCl 0.25%, Agar 0.3%). Plates were incubated for 32 h at 37°C before analysis. Motility ability was observed by measuring the diameter of the motility halo. Non-motile strains grew only at the inoculation point.

### Bacterial adherence assays

Fimbriae- or flagella-mediated binding specificity of C83902 strain and various deletion mutants were determined by cell adhesion assay. The procedure was described previously [11]. Briefly, 1 × 10^7^ CFU of bacteria suspended in PBS containing 4% D-Mannose were added to a monolayer of about 1 × 10^5^ IPEC-J2 cells in each well of a 96-well tissue culture plate (Corning, NY, USA). D-Mannose was used to prevent binding by Type 1 fimbriae [12]. After 1 h incubation, the cell monolayer was gently washed three times with PBS and lysed with 0.5% Triton X-100 for 20 min. The lysates containing total cell-associated bacteria were diluted 1:10 in PBS and plated onto LB agar plates at 37°C for the enumeration of adherent bacteria.

### Adherence inhibition assays

Anti-F4ac polyclonal antiserum was made in our laboratory. Briefly, we constructed a pBR-fae plasmid that could express abundant F4ac fimbriae in *E. coli* strain SE5000. Purified F4ac fimbriae were used to immunize rabbits. The sensitivity and specificity of the polyclonal antiserum was demonstrated using ELISA, agglutination tests and Western blots. This rabbit polyclonal antiserum to F4ac fimbriae at 1:40 was co-incubated with bacterial suspensions for 30 min at 37°C (5% CO_2_) with gentle agitation prior to their addition onto the IPEC-J2 cell monolayer.

### RNA extraction and quantitative fluorescent PCR

Total RNA was extracted from various bacterial samples using TRIzol reagent (Invitrogen, NY, USA) according to the manufacturer’s instruction. RNA quality and quantity were assessed by agarose gel electrophoresis and UV spectrophotometer, respectively. cDNA was synthesized using the PrimeScript^®^RT reagent Kit with gDNA Eraser (Takara Bio, Shiga, Japan) for reverse transcription-PCR. *GapA* was used as an internal control to normalize the threshold cycle (Ct) values of other products. Primer sequences for amplification of *fliC*, *faeG* and *gapA* are listed (Table 1). Real-time PCR amplification followed the guide of SYBR^®^ Premix Ex Taq II (Takara Bio, Shiga, Japan). Assays were performed in quadruplicate with a 7500 Fast Real-Time system (Applied Biosystems), and the dissociation curve was analyzed after amplification. The 2^-△△CT^ method was used for normalizing the relative quantification results [13].

### Statistical analysis

Data were analyzed with SPSS 16 using Student’s *t*-test for independent samples. Differences were considered significant if *P* ≤ 0.05.

## Results

### Morphology and motility of the C83902 strain and isogenic mutants

**Figure 1:**
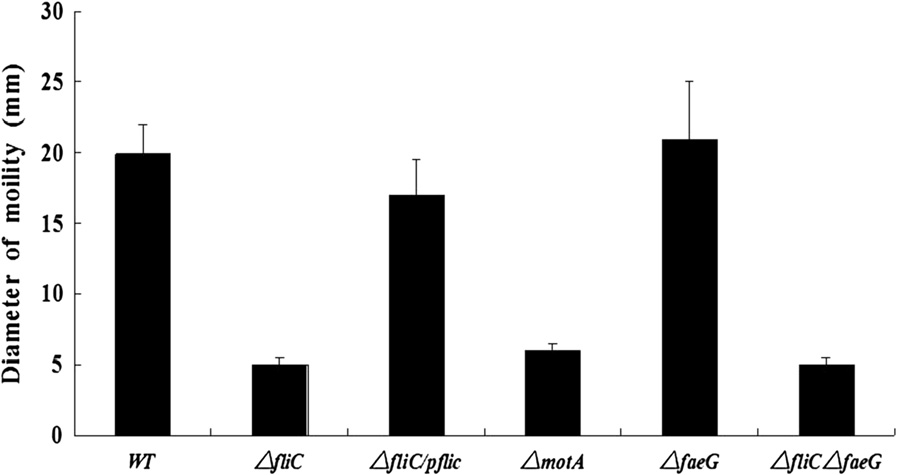
**Swimming motility diameter of WT C83902, isogenic mutants and complemented mutant.** Data represent the averages of three separate experiments. WT, wild-type.

C83902 *E. coli* was chosen as the parent strain to construct the *fliC*, *motA* and *faeG* isogenic single mutants, as well as the *fliC*&*faeG* double mutant. Wild-type (WT) C83902 was motile on semi-solid agar, while the C83902 Δ*fliC*, C83902 Δ*fliC* Δ*faeG* and C83902 Δ*motA* mutants were non-motile (Figure 1). Flagella were not detected in either the *fliC* deletion mutant or the Δ*fliC* Δ*faeG* double mutant by TEM (Data not shown). The complemented strain (C83902 Δ*fliC*/p*fliC*) had restored expression of flagella and motility (Figure 1). The successful construction of the fimbriae deletion mutant (C83902 Δ*faeG*) was confirmed by combined methods of DNA sequencing and an agglutination reaction, as the Δ*faeG* mutant strain lost the ability to agglutinate with the F4 monoclonal antibody.

### C83902 Δ*fliC* mutant decreases the adhesion to IPEC-J2 cells

**Figure 2:**
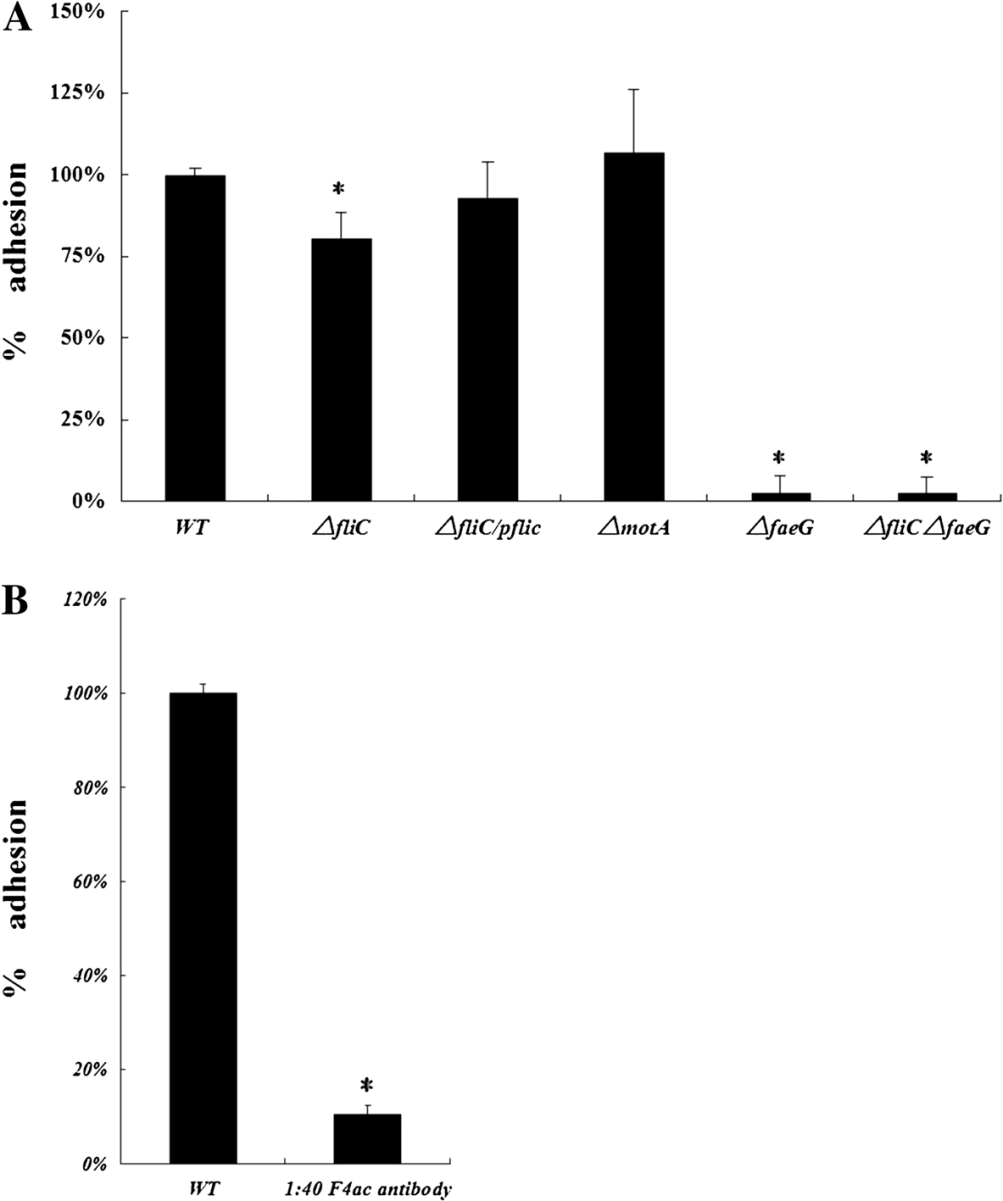
**Both flagella and F4 fimbriae contribute to adherence to IPEC-J2 cells. A**. Flagella is an important F4^+^ ETEC adherence factor. Adherence to IPEC-J2 cells by WT C83902, isogenic mutants and complemented C83902 Δ*fliC*/p*fliC*. **B**. Adherence assay of F4^+^ ETEC to IPEC-J2 cells after pre-incubation with blocking antibody. The WT strain adhesion index was assumed as 100%. Data are expressed as mean ± standard deviation of triplicate experiments. *Indicates statistically significant difference when compared to the WT C83902 strain (*P* < 0.05). WT, wild-type.

It has been reported that the IPEC-J2 cell line is an ideal in vitro model to study F4ac^+^*E. coli* infection [12,14,15]. We found that the C83902 strain could adhere to this cell line. The isogenic C83902 Δ*faeG* mutant had a 97% reduction in adherence as compared with the parent strain (*P* < 0.05, Figure 2A). The Δ*fliC* mutant also showed a 20% reduction in the ability to adhere to IPEC-J2 cells (*P* < 0.05, Figure 2A). Flagellin expression was sufficient to mediate adherence to the IPEC-J2 cells, since the *fliC* complemented strain was able to restore the adherence ability to 94% of WT levels (Figure 2A). By contrast, the adherence ability of the flagellin-expressing but non-motile Δ*motA* mutant was not significantly different from the parent strain (Figure 2A). A 90% reduction in adherence was obtained by incubating IPEC-J2 cells with anti-F4ac antibody (1:40 dilution) (*P* < 0.05, Figure 2B).

### Interaction between ETEC flagella and fimbriae

**Figure 3:**
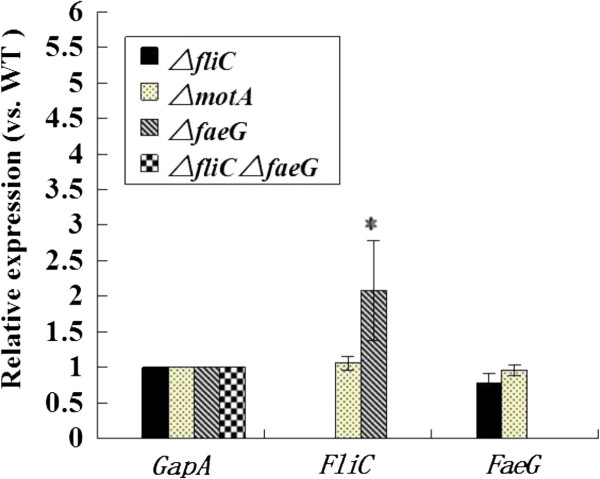
**Expression level of flagella and fimbriae genes in different isogenic mutants compared with WT C83902.***GapA* was used as the normalizing internal standard. The transcriptional expression of the detected genes was measured by qRT-PCR. *Indicates statistically significant difference when compared to the WT C83902 strain (*P* < 0.05). WT, wild-type.

While deleting either *fliC* or *faeG* decreased ETEC adhesion to IPEC-J2 cells, the capacity of the C83902 Δ*fliC* Δ*faeG* double mutant strain to adhere to IPEC-J2 cells was not significantly different from the Δ*faeG* mutant (Figure 2A). To determine whether flagella and fimbriae expression is regulated independently, qRT-PCR was used to quantify the expression of *fliC* and *faeG* in the parent strain and in the various mutants. The expression of *faeG* in the C83902 Δ*fliC* mutant was down regulated by 21.5%, as compared with their expression in the WT strain. By contrast, *fliC and faeG* expression in the Δ*motA* mutant was not significantly different from WT (Figure 3). *fliC* was significantly up-regulated after *faeG* deletion (*P* < 0.05, Figure 3).

## Discussion

F4^+^ ETEC bound avidly to the IPEC-J2 cell line in vitro, which is derived from the jejunum of an un-suckled 1-day-old piglet [12]. It had been reported that the highly conserved regions of ETEC flagellin interact with the two-partner secretion protein A (*EtpA*) to mediate adherence and intestinal colonization [16]. In this study, we show that the capacity of the Δ*fliC* mutant to adhere to IPEC-J2 cells was only 80% of that of the WT C83902 strain. To determine whether the flagellar structure itself possessed an adhesive function or flagellin expression affected other adhesive structures in F4^+^ ETEC, we deleted the *faeG* gene from the parent strain. The significant decrease of adhesion to IPEC-J2 cells and adherence inhibition assay using anti-F4ac antiserum demonstrated that F4ac fimbriae were providing the major adherence factor, similar to what Zhang et al. reported previously [17]. By contrast, treating cells with rabbit anti-FliC polyclonal antiserum, had no impact on F4^+^ ETEC adherence to IPEC-J2 cells, indicating that the flagellin does not directly bind to cells (Data not shown). The mRNA expression of the *faeG* gene decreased 21.5% in the Δ*fliC* mutant compared to the parent strain. We suggest that the 20% reduction of the C83902 Δ*fliC* mutant could be due to the down-regulated expression of F4 fimbriae by the absence of flagellin. qRT-PCR was used to measure the mRNA expression of *faeG* in the *fliC* complemented strain C83902Δ*fliC/pfliC*. *faeG* expression in C83902Δ*fliC/pfliC* was not significantly different from its expression in the WT strain (expression of *faeG* in the complement strain is about 103% in average of the WT), which also confirmed our suggestion.

It has also been demonstrated that flagellum-mediated motility is essential for enhancing pathogen-host interactions and for promoting the subsequent adherence and colonization of several other gram-negative pathogens [18-21]. Surprisingly, there were no differences in the mRNA expression level of above-mentioned flagellin or fimbriae between the highly motile *E. coli* C83902 strain and the non-motile Δ*motA* deletion mutant. The adherence ability of Δ*motA* deletion mutant was also similar to that of the parent strain. These data indicate that motility may be unnecessary for *E. coli* C83902 strain adherence to IPEC-J2 cells in vitro.

This study provides the first evidence that the expression of flagella coordinately regulates the expression of fimbriae in F4^+^ ETEC. From our qRT- PCR data, flagella expression is correlated with the expression of F4 fimbriae. We observed that deletion of flagellin also repressed F4 fimbriae expression. Without F4 fimbriae expression, bacteria can express more flagella, which may directly enhance motility. However,the mechanism by which flagellar deficiency alters F4 fimbriae expression is unclear. One possibility is that there may be a regulatory crosstalk among bacterial surface organelles. Further work is needed to study the coordinated regulation of these surface structures in F4^+^ ETEC.

Johnson et al. reported that the heat-labile enterotoxin promotes *Escherichia coli* adherence to intestinal epithelial cells [1]. It is conceivable that flagellin may regulate some virulence factors such as LT secretion by affecting different types of secretion systems [22,23].

In conclusion, we demonstrated that F4ac^+^ ETEC flagella is a potential virulence factor and its expression is significantly correlated with other virulence factors, such as F4 fimbriae. Flagellin absence down-regulated the expression of F4 fimbriae and decreased the adhesion to IPEC-J2 cells in vitro. However, the in vivo relevance of our findings remains to be determined.

## Competing interests

The authors declare that they have no competing interests.

## Authors’ contributions

GZ, MZ and QD participated in the design of the study. MZ, XZ, ZG and YL performed the experiment. MZ analysed the data and wrote the manuscript. GZ, QD and PRH helped with the revision of this manuscript. All authors read and approved the final manuscript.
